# Emotion Recognition in Immersive Virtual Reality: From Statistics to Affective Computing

**DOI:** 10.3390/s20185163

**Published:** 2020-09-10

**Authors:** Javier Marín-Morales, Carmen Llinares, Jaime Guixeres, Mariano Alcañiz

**Affiliations:** Instituto de Investigación e Innovación en Bioingeniería, Universitat Politècnica de València, 46022 València, Spain; cllinare@omp.upv.es (C.L.); jaiguipr@i3b.upv.es (J.G.); malcaniz@i3b.upv.es (M.A.)

**Keywords:** affective computing, emotion recognition, emotion elicitation, virtual reality, head-mounted display, machine learning

## Abstract

Emotions play a critical role in our daily lives, so the understanding and recognition of emotional responses is crucial for human research. Affective computing research has mostly used non-immersive two-dimensional (2D) images or videos to elicit emotional states. However, immersive virtual reality, which allows researchers to simulate environments in controlled laboratory conditions with high levels of sense of presence and interactivity, is becoming more popular in emotion research. Moreover, its synergy with implicit measurements and machine-learning techniques has the potential to impact transversely in many research areas, opening new opportunities for the scientific community. This paper presents a systematic review of the emotion recognition research undertaken with physiological and behavioural measures using head-mounted displays as elicitation devices. The results highlight the evolution of the field, give a clear perspective using aggregated analysis, reveal the current open issues and provide guidelines for future research.

## 1. Introduction

Emotions play an essential role in rational decision-making, perception, learning and a variety of other functions that affect both human physiological and psychological status [1]. Therefore, understanding and recognising emotions are very important aspects of human behaviour research. To study human emotions, affective states need to be evoked in laboratory environments, using elicitation methods such as images, audio, videos and, recently, virtual reality (VR). VR has experienced an increase in popularity in recent years in scientific and commercial contexts [2]. Its general applications include gaming, training, education, health and marketing. This increase is based on the development of a new generation of low-cost headsets which has democratised global purchases of head-mounted displays (HMDs) [3]. Nonetheless, VR has been used in research since the 1990s [4]. The scientific interest in VR is due to the fact that it provides simulated experiences that create the sensation of being in the real world [5]. In particular, environmental simulations are representations of physical environments that allow researchers to analyse reactions to common concepts [6]. They are especially important when what they depict cannot be physically represented. VR makes it possible to study these scenarios under controlled laboratory conditions [7]. Moreover, VR allows the time- and cost-effective isolation and modification of variables, unfeasible in real space [8].

### 1.1. Virtual Reality Set-Ups

The set-ups that display VR simulations have been progressively integrated into studies as the relevant technologies have evolved. These consist of a combination of three objective features, formats, display devices and user interfaces.

The format describes the structure of the information displayed. The most common are two-dimensional (2D) multimedia and three-dimensional (3D) environments, and the main difference between them is their levels of interactivity [9]. 2D multimedia, including 360° panoramic images and videos, provide non-interactive visual representations. The validity of this format has been extensively explored [10]. Moreover, the latest advances in computer-generated images simulate light, texture and atmospheric conditions to such a degree of photorealism that it is possible to produce a virtual image that is indistinguishable, to the naked eye, from a photograph of a real-world scene [11]. This format allows scientists to test static computer-generated environments, with many variations, cheaply and quickly in a laboratory. On the other hand, 3D environments generate interactive representations which allow changes in the user’s point of view, navigation and even interaction with objects and people [12]. Developing realistic 3D environments is more time consuming than developing 360° computer-generated photographs, and their level of realism is limited by the power of the hardware. However, the processing potency of GPUs (graphics processing units) is increasing every year, which will enhance the performance of 3D environments. Moreover, the interaction capacity of 3D environments, which facilitates the simulation of real-world tasks, is a key aspect in the application of virtual reality [2].

The display devices are the technological equipment used to visualise the formats. They are classified according to the level of immersion they provide, that is, the sensorimotor contingencies that they support. These are related to the actions that experimental subjects carry out in the perception process, for example, when they bend down and shift the position of their heads, and their gaze direction, to see underneath an object. Therefore, the sensorimotor contingencies supported by a system define a set of valid actions (e.g., turning the head, bending forward) that carry meaning in terms of perception within the virtual environment [13]. Since immersion is objective, one system is more immersive than another if it is superior in at least one characteristic while others remain equal. There are three categories of immersion system, non-immersive, semi-immersive and immersive [2]. Non-immersive systems are simpler devices which use a single screen, such as a desktop PC, to display environments [14]. Semi-immersive systems, such as the cave automatic virtual environment (CAVE), or the powerwall screen, use large projections to display environments on walls, enveloping the viewer [15,16]. These displays typically provide a stereo image of an environment, using a perspective projection linked to the position of the observer’s head. Immersive devices, such as HMDs, are fully-immersive systems that isolate the user from external world stimuli [17]. These provide a complete simulated experience, including a stereoscopic view, which responds to the user’s head movements. During the last two decades, VR has usually been displayed through desktop PCs or semi-immersive systems, such as CAVEs and powerwalls [18]. However, improvements in the performance and availability of the new generation of HMDs is boosting their use in research [19].

The user interfaces, which are exclusive to 3D environments which allow this level of interaction, are the functional connections between the user and the VR environment which allow him or her to interact with objects and navigate [20]. Regarding interaction with objects, manipulation tasks include: selection, that is, acquiring or identifying an object or subset of objects, positioning, that is, changing an object’s 3D position, and rotation, that is, changing an object’s 3D orientation. In terms of the navigation metaphors in 3D environments, virtual locomotion has been thoroughly analysed [21], and can be classified as physical or artificial. Regarding the physical, there are room-scale-based metaphors, such as real-walking, which allow the user to walk freely inside a limited physical space. These are normally used with HMDs, and position and orientation are determined by the position of the user’s head. They are the most naturalistic of the metaphors, but are highly limited by the physical tracked area [22]. In addition, there are motion-based metaphors, such as walking-in-place or redirected walking. Walking-in-place is a pseudo-naturalistic metaphor where the user performs a virtual locomotion to navigate, for example, by moving his/her hands as if (s)he was walking, or by performing footstep-like movements, while remaining stationary [23]. Redirected walking is a technique where the user perceives (s)he is walking freely but, in fact, is being unknowingly manipulated by the virtual display: this allows navigation in an environment larger than the actual tracked area [24]. Regarding the artificial, controller-based metaphors allow users to control their movements directly through joysticks or similar devices, such as keyboards and trackballs [25]. In addition, teleportation-based metaphors allow the user to point where (s)he wants to go and teleport him or her there with an instantaneous “jump” [26]. Moreover, recent advancements in the latest generation HMD devices have increased the performance of navigation metaphors. Point-and-click teleport metaphors have become mainstream technologies implemented in all low-cost devices. However, other techniques have also increased in performance: walking-in-place metaphors have become more user-friendly and robust, room-scale-based metaphors now have increased coverage areas, provided by low-cost tracking methods, and controller-based locomotion now addresses virtual sickness through effective, dynamic field-of-view adjustments [27].

### 1.2. Sense of Presence

In addition to the objective features of the set-up, the experience of users in virtual environments can be measured by the concept of presence, understood as the subjective feeling of “being-there” [28]. A high degree of presence creates in the user the sensation of physical presence and the illusion of interacting and reacting as if (s)he was in the real world [29]. In the 2000s, the strong illusion of being in a place, in spite of the sure knowledge that one is not actually there, was characterised as “place illusion” (PI), to avoid any confusion that might be caused by the multiple meanings of the word “presence”. Moreover, just as PI relates to how the world is perceived, and the correlation of movements and concomitant changes in the images that form perceptions, “plausibility illusion” (PsI) relates to what is perceived, in a correlation of external events not directly caused by the participant [13]. PsI is determined by the extent to which a system produces events that directly relate to the participant, and the overall credibility of the scenario being depicted in comparison with viewer expectations, for example, when an experimental participant is provoked into giving a quick, natural and automatic reply to a question posed by an avatar.

Although presence plays a critical role in VR experiences, there is limited understanding of what factors affect presence in virtual environments. However, there is consensus that exteroception and interoception factors affect presence. It has been shown that exteroception factors, such as higher levels of interactivity and immersion, which are directly related to the experimental set-up, provoke increased presence, especially in virtual environments not designed to induce particular emotions [30,31,32]. As to the interoception factors, which are defined by the content displayed, participants will perceive higher presence if they feel emotionally affected; for example, previous studies have found a strong correlation between arousal and presence [33]. Recent research has also analysed presence in specific contexts and suggested that, for example, in social environments, it is enhanced when the VR elicits genuine cognitive, emotional and behavioural responses, and when participants create their own narratives about events [34]. On the other hand, presence decreases when users experience physical problems, such as cybersickness [35].

### 1.3. Virtual Reality in Human Behaviour Research

VR is, thus, proposed as a powerful tool to simulate complex, real situations and environments, offering researchers unprecedented opportunities to investigate human behaviour in closely controlled designs in controlled laboratory conditions [33]. There are now many researchers in the field, who have published many studies, so a strong, interdisciplinary community exists [2].

Education and training is one field where VR has been much applied. Freina and Ott [36] showed that VR can offer great educational advantages. It can solve time-travel problems, for example, students can experience different historical periods. It can address physical inaccessibility, for example, students can explore the solar system in the first person. It can circumnavigate ethical problems, for example, students can “perform” serious surgery. Surgical training is now one of the most analysed research topics. Interventional surgery lacked satisfactory training methods before the advent of VR, except learning on real patients [37]. Bhagat, Liou and Chang [38] analysed improvements in military training. These authors suggested that cost-effective 3D VR significantly improved subjects learning motivation and outcomes and provided a positive impact on their live-firing achievement scores. In addition, besides enhancements in cost-effectivity, VR offers a safe training environment, as evidenced by the extensive research into driving and flight simulators [39,40]. Moreover, de-Juan-Ripoll et al. [41] proposed that VR is an invaluable tool for assessing risk-taking profiles and to train in related skills, due to its transferability to real-world situations.

Several researchers have also demonstrated the effectiveness of VR in therapeutic applications. It offers some distinct advantages over standard therapies, including precise control over the degree of exposure to the therapeutic scenario, the possibility of tailoring scenarios to individual patients’ needs and even the capacity to provide therapies that might otherwise be impossible [42]. Taking some examples, studies using VR have analysed the improvement in the training in social skills for persons with mental and behavioural disorders, such as phobias [43], schizophrenia [44] and autism [45]. Lloréns, Noé, Colomer and Alcañiz [46] showed that VR-based telerehabilitation interventions promoted the reacquisition of locomotor skills associated with balance, in the same way as in-clinic interventions (both complemented with conventional therapy programmes). Moreover, it has been proposed as a key tool for the diagnosis of neurodevelopmental disorders [47].

In addition, VR has been applied transversally to many fields, such as architecture and marketing. In architecture, VR has been used as a framework within which to test the overall validity of proposed plans and architectural designs, generate alternatives and conceptualise learning, instruction and the design process itself [48]. In marketing, it has been applied in the analysis of consumer behaviour in laboratory-controlled conditions [49] and as a tool to develop emotionally engaging consumer experiences [50].

One of the most important topics in human behaviour research is human emotions, due to the central role that they play in many background processes, such as perception, decision-making, creativity, memory and social interaction [51]. Given the presence that VR provokes in users, it has been suggested as a powerful means of evoking emotions in laboratory environments [8]. In one of the first confirmatory studies into the efficacy of immersive VR as an affective medium, Baños et al. [30] showed that emotion has an impact on presence. Subsequently, many other similar studies showed that VR can evoke emotions, such as anxiety and relaxation [52], positive valence in obese children taking exercise [53], arousal in natural environments, such as parks [54], and different moods in social environments featuring avatars [55].

### 1.4. The Validity of Virtual Reality

Finally, it is crucial to point out that the usefulness of simulation in human behaviour research has been analysed through the validity concept, that is, the capacity to evoke a response from the user in a simulated environment similar to one that might be evoked by a physical environment [56]. Thus, there is a need to perform direct comparisons between virtual and real environments. Some comparisons have studied the validity of virtual environments by assessing psychological responses [57] and cognitive performance [58]. However, there have been fewer analyses of physiological and behavioural responses [59,60]. Heydarian et al. analysed user performance in office-related activities, for example, reading texts and identifying objects, and found that the participants performed similarly in an immersive virtual environment setting and in a benchmarked physical environment for all of the measured tasks [61]. Chamilothori, Wienold, and Andersen compared subjective perceptions of daylit spaces, and identified no significant differences between the real and virtual environments studied [62]. Kimura et al. analysed orienteering-task performance, where participants in a VR room showed less facility, suggesting that caution must be applied when interpreting the nuances of spatial cue use in virtual environments [63]. Higuera-Trujillo, López-Tarruella, and Llinares analysed psycho-physiological responses, through electrodermal activity (EDA), evoked by real-world and VR scenarios with different immersion levels, and demonstrated correlations in the physiological dynamics between real-world and 3D environments [64]. Marín-Morales et al. analysed the emotional responses evoked in subjects in a real and a virtual museum, and found no self-assessment differences, but did find differences in brain dynamics [65]. Therefore, further research is needed to understand the validity of VR in terms of physiological responses and behavioural performance.

### 1.5. Implicit Measures and the Neuroscience Approach

Traditionally, most theories of human behaviour research have been based on a model of the human mind that assumes that humans can think about and accurately verbalise their attitudes, emotions and behaviours [66]. Therefore, classical psychological evaluations used self-assessment questionnaires and interviews to quantify subjects’ responses. However, these explicit measures have been demonstrated to be subjective, as stereotype-based expectations can lead to systematically biased behaviour, given that most individuals are motivated to be, or appear to be, nonbiased [67]. The terms used in questionnaires can also be differentially interpreted by respondents, and the outcomes depend on the subjects possessing a wide knowledge of their dispositions, which is not always the case [68].

Recent advances in neuroscience show that most of the brain processes that regulate our emotions, attitudes and behaviours are not conscious. In contrast to explicit processes, humans cannot verbalise these implicit processes [69]. In recent years, growing interest has developed in “looking” inside the brain to seek solutions to problems that have not traditionally been addressed by neuroscience. Thus, neuroscience offers techniques that can recognise implicit measurements not controlled by conscious processes [70]. These developments have provoked the emergence in the last decades of a new field called neuroeconomics, which blends psychology, neuroscience and economics into models of decision-making, rewards, risks and uncertainties [71]. Neuroeconomics addresses human behaviour research, in particular the brain mechanisms involved in economic decision-making, from the point of view of cognitive neuroscience, using implicit measures.

Several implicit measuring techniques have been proposed in recent years. Some examples of their applications in human behaviour research are: heart rate variability (HRV) has been correlated with arousal changes in vehicle drivers when detecting critical points on a route [72], electrodermal activity (EDA) has been used to measure stress caused by cognitive load in the workplace [73], electroencephalogram (EEG) has been used to assess engagement in audio-visual content [74], functional magnetic resonance imaging (fMRI) has been used to record the brain activity of participants engaged in social vs. mechanical/analytic tasks [75], functional near-infrared spectroscopy (fNIRS) has been used as a direct measure of brain activity related to decision-making processes in approach-avoidance theories [76], eye-tracking (ET) has been used to measure subconscious brain processes that show correlations with information processing in risky decisions [77], facial expression analysis (FEA) has been applied to detect emotional responses in e-learning environments [78] and speech emotion recognition (SER) has been used to detect depressive disorders [79]. Table 1 gives an overview of the implicit measuring techniques that have been used in human behaviour research.

In addition, recent studies have highlighted the potential of virtual reality environments for enhancing ecological validity in the clinical, affective and social neurosciences. These studies have usually involved the use of simple, static stimuli which lack many of the potentially important aspects of real-world activities and interactions [90]. Therefore, VR could play an important role in the future of neuroeconomics by providing a more ecological framework within which to develop experimental studies with implicit measures.

### 1.6. Affective Computing and Emotion Recognition Systems

Affective computing, which analyses human responses using implicit measures, has developed into an important field of study in the last decades. Introduced by Rosalind Picard in 1997, it proposed the automatic quantification and recognition of human emotions as an interdisciplinary field based on psychophysiology, computer science, biomedical engineering and artificial intelligence [1]. The automatic recognition of human emotion statements using implicit measures can be transversally applied to all human behaviour topics and complement classic explicit measures. In particular, it can be applied to neuroeconomic research as they share the same neuroscientific approach of using implicit measures, and due to the important relationship that has been found between emotions and decision-making [71]. Emotion recognition models can be divided into three approaches: emotional modelling, emotion classification and emotion elicitation.

The emotional modelling approach can be divided into the discrete and the dimensional. Discrete models characterise the emotion system as a set of basic emotions, which includes anger, disgust, fear, joy, sadness and surprise, and the complex emotions that result from combining them [91]. On the other hand, dimensional models propose that emotional responses can be modelled in a multidimensional space where each dimension represents a fundamental property common to all emotions. The most commonly used theory is the circumplex model of affect (CMA), which proposes a three-dimensional space consisting of: valence, that is, the degree to which an emotion is perceived as positive or negative, arousal, that is, the intensity of the emotion in terms of activation, from low to high, and dominance, which ranges from feelings of total lack of control or influence on events and surroundings to the opposite extreme of feeling influential and in control [92].

Affective computing uses biometric signals and machine-learning algorithms to classify emotions automatically. Many signals have been used, such as voice, face, neuroimaging and physiological [93]. It is noteworthy that one of the main emotion classification topics uses variables associated with central nervous system (CNS) and autonomic nervous system (ANS) dynamics [93]. First, human emotional processing and perception involve cerebral cortex activity, which allows the automatic classification of emotions using the CNS. EEG is one of the techniques most used in this context [94]. Second, many emotion recognition studies have used the ANS to analyse the changes in cardiovascular dynamics provoked by mood changes, where HRV and EDA are the most used techniques [95]. The combination of physiological features and machine-learning algorithms, such as in support vector machines, linear discriminant analysis, K-nearest neighbour and neural networks, has achieved high levels of accuracy in inferring subjects’ emotional states [96].

Finally, emotion elicitation is the ability to reliably and ethically elicit affective states. This elicitation is a critical factor in the development of systems that can detect, interpret and adapt to human affect [97]. The many methods that elicit emotions in laboratories can be mainly divided into two groups, active and passive. Active methods involve directly influencing subjects, including behavioural manipulation [98], social interaction [99] and dyadic interaction [100]. Passive methods usually present external stimuli, such as images, sound or video. As to the use of images, the International Affective Picture System (IAPS) is among the databases most used as an elicitation tool in emotion recognition methodologies [95]. This includes over a thousand depictions of people, objects and events, standardised on the basis of valence and arousal [97]. As to audio, the International Affective Digitalised Sound System (IADS) database is the most commonly applied in studies which use sound to elicit emotions [101]. However, some studies directly use music or narrative to elicit emotions [102]. With respect to audio-visual stimuli, many studies have used film to induce arousal and valence [103]. These emotion elicitation methods have two important limitations. The set-ups used, mostly screens, are non-immersive devices, which provoke only a low level of presence in subjects [30]. Therefore, the stimuli do not evoke in the subjects a feeling of “being there”, which is needed to analyse emotions in simulated real-world situations. In addition, the stimuli are non-interactive, so they do not allow the subjects to intervene in the scene, which would open the possibility to recognise emotional states during interactive tasks. These limitations can be overcome by using immersive VR as a new emotion elicitation method. Since the year 2000, VR has increasingly been used as affective stimulation, however the majority of the studies undertaken have applied classic statistical methods, such as hypotheses testing and correlation, to analyse subjects’ physiological responses to different emotions [104]. However, in recent years, some research has started to apply affective computing paradigms with VR as the emotion elicitation method, combining implicit measures with machine-learning methods to develop automatic emotion recognition models [105].

This paper provides a systematic review of the literature on the use of head-mounted displays in implicit measure-based emotion recognition research, and examines the evolution of the research field, the emotions analysed, the implicit techniques, the data analysis, the set-ups and the validations performed.

## 2. Materials and Methods

### Data Collection

We followed an adapted version of the Preferred Reporting Items for Systematic Reviews and Meta-Analyses (PRISMA) study selection guidelines [106]. This includes steps to identify literature, to screen the identified literature, to check the eligibility of the screened literature and, finally, to synthesise the literature. The screening and eligibility steps were performed simultaneously. The literature search was carried out on 25 March 2020. The Scopus database was queried using the following search string: TITLE-ABS-KEY (“virtual reality” OR “head-mounted display”) AND TITLE-ABS-KEY (“emotion*” OR “affective*”) AND DOCTYPE (ar OR re). The keywords virtual reality OR head-mounted display include all the studies on VR and, in particular, all that used HMDs. In addition, the keywords emotion* OR affective* include all the papers related to emotion. The combination of both requirements revealed the research that included virtual reality and emotions. The search was limited to articles in journals and reviews (for snowballing). A total of 1424 records were identified. Some 14 additional records were identified from other sources.

**Figure 1:**
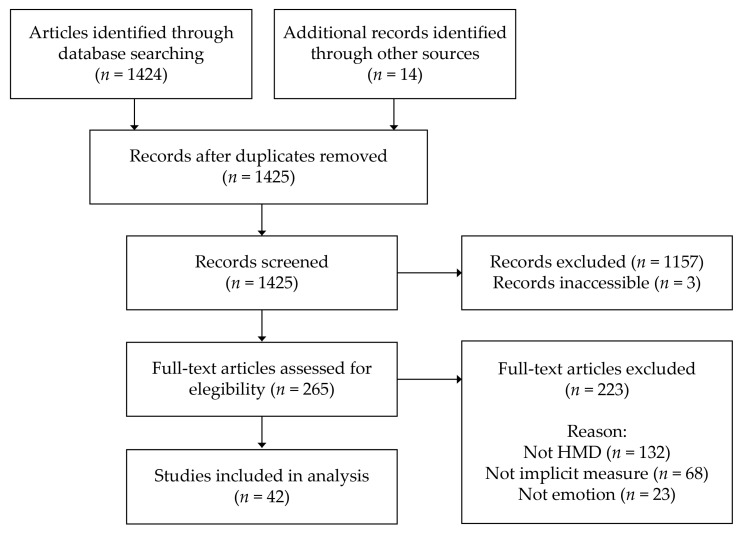
Scheme of the PRISMA procedure followed in the review.

The screening and eligibility checks were undertaken as follows: (1) first, by investigating titles and abstracts, 13 duplicates were identified. (2) The manuscripts were superficially screened for a thematic match with virtual reality as emotion elicitation. A total of 1157 records were excluded for not matching with the topic, and 3 records because they were inaccessible. (3) We investigated 265 records to exclude those that did not fit, using a specific rejection order: that is, if they used HMDs, we moved on to the next filter criterion, implicit measures, if they used implicit measures, we moved on to the last criterion, the analysis of an emotion. Some 132 records were rejected for not using HMDs, 68 for not using implicit measures and 23 for not analysing an emotional dimension. Finally, 42 studies were included in the analysis which used virtual reality displayed in an HMD, in combination with any implicit measure to analyse or recognise emotional states. The summary of the procedure is depicted in Figure 1.

## 3. Results

### 3.1. Summary of Previous Research

In recent years, studies have applied implicit measures to analyse emotions using immersive VR with HMDs. Table 2 provides a summary of the studies included in the analysis.

### 3.2. Evolution of the Research

**Figure 2:**
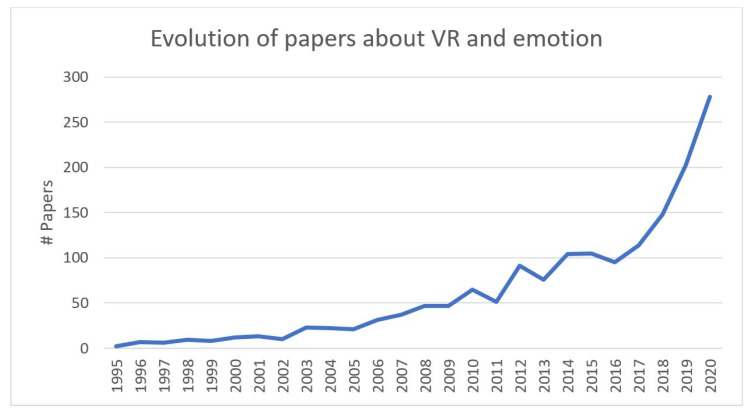
Evolution of the number of papers published each year on the topic of virtual reality and emotions. The total number of papers to be published in 2020 has been extrapolated using data up to 25 March 2020.

Figure 2 shows the number of papers published each year which included the topics virtual reality and emotion analysis. This number of studies was calculated based on all the papers screened. In the 1990s, the average number of papers published annually was 6.4, the first being published in 1995. In the 2000s, the average number of papers published increased to 26.3. However, from 2010 to 2014, the average multiplied by three to 77.4. In the last five years, the curve has grown exponentially to 203 in 2019, and a predicted 278 in 2020.

### 3.3. Emotions Analysed

**Figure 3:**
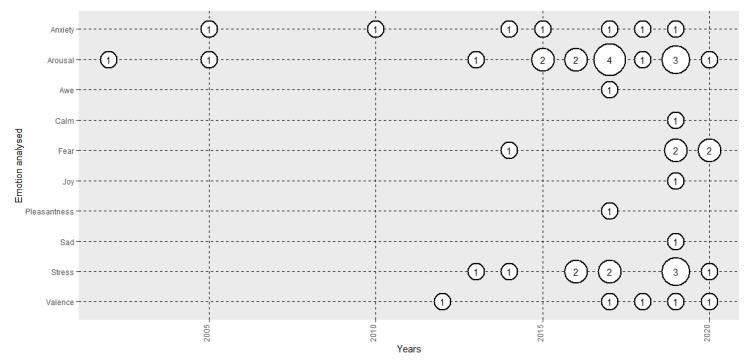
Evolution of the number of papers published each year based on emotion analysed.

Figure 3 depicts the evolution in the number of papers analysed in the review based on the emotion under analysis. Until 2015, the majority of the papers analysed arousal-related emotions, mostly arousal, anxiety and stress. From that year, some experiments started to analyse valence- related emotions, such as valence, joy, pleasantness and sadness, but the analysis of arousal-related emotions still predominated. Some 50% of the studies used CMA (arousal 38.1% [54] and valence 11.9% [125]), and the other 50% used basic or complex emotions (stress 23.8% [112], anxiety 16.7% [109], fear 11.9% [43], awe 2.4% [121], calmness 2.4% [135], joy 2.4% [135], pleasantness 2.4% [64] and sadness 2.4% [135]).

### 3.4. Implicit Technique, Features used and Participants

**Figure 4:**
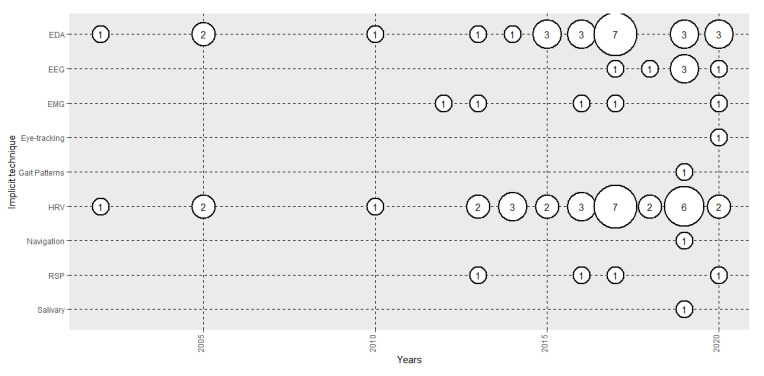
Evolution of the number of papers published each year based on the implicit measure used.

Figure 4 shows the evolution of the number of papers analysed in terms of the implicit measures used. The majority used HRV (73.8%) and EDA (59.5%). Therefore, the majority of the studies used ANS to analyse emotions. However, most of the studies that used HRV used very few features from the time domain, such as HR [115,120]. Very few studies used features from the frequency domain, such as HF, LF or HF/LF [119,126] and 2 used non-linear features, such as entropy and Poincare [65,105]. Of the studies that used EDA, the majority used total skin conductance (SC) [116], but some used tonic (SCL) [54] or phasic activity (SCR) [124]. In recent years, EEG use has increased, with 6 papers being published (14.3%), and the CNS has started to be used, in combination with HMDs, to recognise emotions. The analyses that have been used are ERP [138], power spectral density [140] and functional connectivity [65]. EMG (11.9%) and RSP (9.5) were also used, mostly in combination with HRV. Other implicit measures used were eye-tracking, gait patterns, navigation and salivary cortisol responses. The average number of participants used in the various studies depended on the signal, that is, 75.34 (σ = 73.57) for EDA, 68.58 (σ = 68.35) for HRV and 33.67 (σ = 21.80) for EEG.

### 3.5. Data Analysis

**Figure 5:**
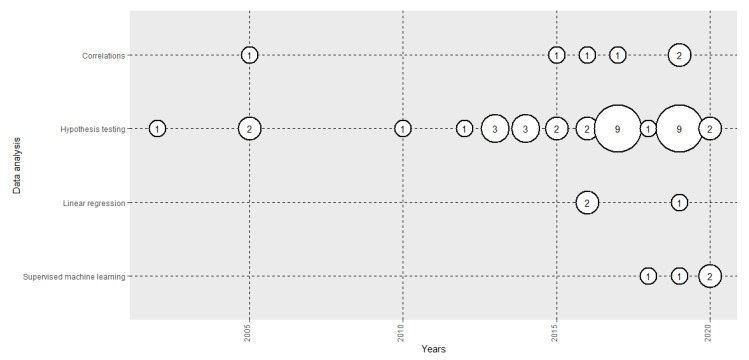
Evolution of the number of papers published each year by data analysis method used.

Figure 5 shows the evolution of the number of papers published in terms of the data analysis performed. The vast majority analysed the implicit responses of the subjects in different emotional states using hypothesis testing (83.33%), correlations (14.29) or linear regression (4.76%). However, in recent years, we have seen the introduction of applied supervised machine-learning algorithms (11.90%), such as SVM [105], Random Forest [139] and kNN [140] to perform automatic emotion recognition models. They have been used in combination with EEG [65], HRV [105] and EDA [140].

### 3.6. VR Set-Ups Used: HMDs and Formats

**Figure 6:**
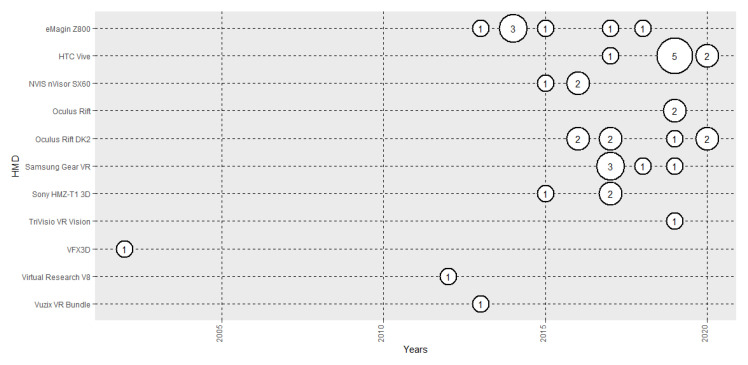
Evolution of the number of papers published each year based on head-mounted display (HMD) used.

Figure 6 shows the evolution of the number of papers published based on HMD used. In the first years of the 2010s, eMagin was the most used. In more recent years, advances in HMD technologies have positioned HTC Vive as the most used (19.05%). In terms of formats, 3D environments are the most used [138] (85.71%), with 360° panoramas following far behind [142] (16.67%). One research used both formats [64].

### 3.7. Validation of VR

Table 3 shows the percentage of the papers that presented analyses of the validation of VR in an emotional research. Some 83.33% of the papers did not present any type of validation. Three papers included direct comparisons of results between VR environments and the physical world [64,65,109], and 3 compared, in terms of the formats used, the emotional reactions evoked in 3D VRs, photos [109], 360° panoramas [64] and augmented reality [129]. Finally, another compared the influence of immersion [121], the similarity of VR results with previous datasets [108] and one compared its results with a previous version of the study performed in the real world [132].

## 4. Discussion

This work highlights the evolution of the use of immersive VR, in particular using head-mounted displays, in emotion recognition research in combination with implicit measures. It provides a clear perspective based on a systematic review and aggregated analysis, focusing on the role that VR might play as an emotion elicitation tool in the coming years.

The evolution of scientific interest in VR and emotions has grown exponentially, to more than 200 papers per year (Figure 2). In particular, the performance improvements in the last few years in the latest generation of HMDs, in terms of resolution, field of view, immersion levels and the fall in their price, has boosted their use in emotion-related research. This accords with VR’s increased application in recent years in other areas, such as rehabilitation, neurosurgery and therapy [2]. Therefore, the results suggest that the 2010s was the decade of the rapid growth of VR in emotion research using implicit measures, and the 2020s might be the decade when the field matures. Environmental simulations might, in the future, normally go beyond the paradigm of non-immersive/video-based 2D images to immersive VR scenarios, where subjects feel a very strong sense of presence and can interact with the stimuli presented.

In regard to HMDs and implicit measures in emotion analysis, there is no consensus about the use of CMA [92] or the Ekman theory of basic emotions [91], since both approaches are used in 50% of the research (Figure 3). The differences in the frameworks used causes some difficulties in comparing the results of different studies. The majority of the studies (90.5%) included analyses of arousal [54], or high-arousal-related discrete emotions, such as stress [112], anxiety [109] and fear [43]. On the other hand, only 23.9% of the studies analysed valence, or discrete emotions closely related to valence, such as awe [121], calm [135], joy [135], pleasantness [64] and sadness [135]. Therefore, although the whole sub-field of affective computing using HMDs is still in its first growth phase, valence recognition and its physiological dynamics, in particular, are under-researched. Recent research since 2017 has started to address this [65,139]. Dominance, a dimension of the CMA still not addressed in general affective computing research using pictures or videos [143], has also not been analysed in HMD set-up research. However, fear, a basic emotion closely related to the dominance dimension, was analysed in 11.9% of the studies examined in the review. In contrast to the fear that is felt when someone watches a horror film, which is based on the empathy of the viewer with the protagonist, the level of presence that immersive VR offers allows the analysis of fear directly felt by subjects based on scenarios they are viewing. Therefore, VR can boost the analysis of the dominance dimension in affective computing in the future. In addition, VR allows researchers to analyse emotional reactions to social stimuli, such as avatars [138], which might be the next stage in the application of classic 2D affective computing paradigms to simulated real-world situations, which can provide new insights with a social dimension.

In terms of the implicit techniques used to recognise emotions evoked through HMDs, ANS measurements are most used: specifically, HRV (73.8%) and EDA (59.5%), many times used in combination. However, until 2016, the majority of the papers featured only HR and SC (Table 2), sometimes in combination with EMG and RSP. From 2016, the research started to include HRV frequency domain and non-linear domain analyses [105,119], and EDA analyses, such as CDA, dividing the signals into tonic and phasic components [64]. In terms of the CNS, EEG research has been undertaken since 2016, including ERP [138], power spectral density [140] and functional connectivity analysis [65]. Other non-physiological implicit measures have been used since 2019, such as eye-tracking [141], gait patterns [135], navigation [133] and salivary cortisol responses [132]. The use of behavioural measures, such as eye-tracking, gait patterns and navigation, might be a very powerful approach where VR can contribute to affective computing research, as they provide high levels of interactivity with the simulated stimuli. This might open a new sub-field where emotional states can be assessed through behavioural measures in interactive, real situations.

However, the current weakest point of HMD-based emotion recognition systems is that only 11.90% of the studies, that is, four, used machine-learning algorithms to classify the emotions analysed. Since the early 2000s, when physiological signals, in combination with HMDs, were first applied to analyse emotions, until 2018, all studies used hypothesis testing and/or correlations to provide insights into the ANS oscillations produced during different affective states, except Reference [125], which used EEG. Although the classic statistical techniques obtained important and useful insights, they have some limitations: (i) hypothesis testing analyses differences between two populations based on means and deviations, but does not provide emotion recognition, (ii) it is difficult to analyse the effect of the combination of several features in datasets with large sets of variables and (iii) they do not take into account non-linear relationships. These limitations are being overcome with the use of machine-learning algorithms, as they can recognise emotions through the development of algorithms in classification problems, automatic feature selection procedures to recognise complex patterns inside data and offer non-linear kernels [143]. Marín-Morales et al. [105] presented the first emotion recognition system using SVM in combination with a large set of HRV features (time, frequency and non-linear domains) and EEG (PSD and mean phase coherence) in 360° emotional rooms, achieving a recognition rate of 75% in arousal and 71.21% in valence. Marín-Morales et al. [65] developed an emotion recognition system in a realistic 3D virtual museum, using SVM in combination with HRV and EEG, with rates of 75% and 71.08% of recognition in arousal and valence, respectively. Granato et al. [139] presented an arousal-valence emotion recognition model with subjects playing a VR racing game. This procedure collected physiological responses, that is, EDA, HRV, EMG and RSP. Bălan et al. [140] analysed the performance of a set of machine-learning and deep-learning techniques (kNN, SVM, RF, LDA, NN), which adapted their stimuli based on the level of fear recognised, in fear recognition in a 3D acrophobia game. The results showed recognition levels ranging from 42.5% to 89.5%. Therefore, the development of emotion recognition models in immersive VR is an open, fast-growing sub-field, which is moving from the classic statistical testing paradigm to supervised machine-learning.

As to the set-ups employed, Figure 6 shows the evolution of the HMDs used in implicit measure-based emotion research. Among the first-generation VR HMDs of the 2000s was VFX3D, which offers a resolution of 380 × 337 per eye. In the 2010s, the eMaginZ800 improved on the resolution of previous HMDs, offering 800 × 600 and 40° of field of view, followed by Oculus Rift DK2, which increased the resolution to 1080 × 960 and, in particular, the FOV to 90°. Finally, in the late 2010s, the HTC Vive offered an increase in resolution to 1600 × 1400 per eye, and democratised VR with its competitive price. Those increments in HMD performance are aligned with the exponential growth of the number of papers that have used HMD in emotion recognition research (Figure 2), and future HMDs, that might achieve 4K of resolution per eye, could boost the use of VR as a tool to recreate real situations in controlled laboratory environments.

The format most used overall was the 3D environment (85.71%)—360° panoramas were used in 16.67% of cases. This is probably due to the fact that 3D environments present a high level of interactivity, as 360° panoramas do not allow changes in point of view. However, both formats can be useful, depending on the aim of the experiment. The 360° panorama set-ups can be very effective for updating classic, closely controlled affective computing methodologies, in particular, when presenting users with a series of non-interactive stimuli, such as IAPS [95] and IADS [144], but increasing degrees of presence based on immersion level [30]. However, there is still a need to develop large datasets of validated immersive stimuli that cover a wide range of emotions, which could be used as general benchmarks to analyse physiological and behavioural dynamics in immersive VR. The 360° approach offers a good solution to this, as the interaction, for example, navigation, provokes uncontrolled variations during the emotional experience. The first dataset of stimuli published was by Marín-Morales et al. [105], which included 4 scenarios that recreated all quadrants of the CMA. On the other hand, the level of interactivity that 3D scenarios offer can be very useful in applied research, since they display more naturalistic and interactive environments, facilitating decision-making research and the analysis of daily situations. Taking some examples, Takac et al. [137] analysed the anxiety felt by speakers when faced by large audiences, Lin et al. [133] analysed the stress felt by individuals when in a building on fire scenario and Kisker et al. [130] analysed arousal in an exposure to a high height.

Immersive VR can be a very powerful tool to analyse human behaviour in controlled laboratory conditions, but we do not yet know the level of VR validity needed to allow the extrapolation to the real world of the insights gained in terms of physiological and behavioural responses. Indeed, 83.33% of the papers did not present any validation, and only 3 provided a direct comparison between the VR scene and the physical environment simulated. Gorini et al. [109] analysed anxiety through HRV and EDA with virtual and real food, Higuera-Trujillo et al. [64] analysed pleasantness through EDA responses in a 3D, 360° and real retail store, and Marín-Morales et al. [65] analysed arousal and valence oscillations with HRV and EEG in a virtual and physical museum. Other research analysed the influence of immersion [121] and other VR features. Thus, VR validation is still an open topic that needs to be more actively addressed. Understanding and isolating the intrinsic dynamics of VR will be key in future years for the validation of the insights obtained using HMDs.

Finally, the results suggest that VR will play a central role in the affective computing field. The research performed has increased its complexity and maturity during the last two decades, and this tendency is likely to continue during the next years. First, future research should extend the analysis of the physiological dynamics using VR as emotion elicitation in VR, to achieve a level of understanding at least as high as we have today using 2D pictures as stimulation. Subsequently, VR might open up many research opportunities that would be very difficult to assess with non-immersive stimuli. In particular, the inclusion of the dominance dimension, which is very closely related to the users’ control of the environment, and impacts on very important features, such as sense of security. Moreover, the social dimension is a crucial factor in the understanding of the emotional dynamics of human beings. The future inclusion of responsive, realistic avatars will help increase the understanding of emotions evoked during social interactions, and the associated physiological responses, in controlled conditions.

## 5. Conclusions

This work analysed the current state-of-the-art in implicit measure-based emotion recognition elicited by HMDs, and gave a perspective using a systematic and aggregated analysis that can guide future research. After two decades of little research analysing emotions using HMDs in combination with implicit measures, mostly undertaken through the physiological arousal responses of the ANS, in recent years, an inflexion point has been reached. The number of papers published is increasing exponentially, and more emotions are being analysed, including valence-related states, more complex biomedical signal processing procedures are increasingly being performed, including EEG analyses and other behavioural measures, and machine-learning algorithms are being newly applied to develop automatic emotion recognition systems. The results suggest that VR might revolutionise emotion elicitation methods in laboratory environments in the next decade, and impact on affective computing research, transversely in many areas, opening new opportunities for the scientific community. However, more research is needed to increase the understanding of emotion dynamics in immersive VR and, in particular, its validity in performing direct comparisons between simulated and real environments.

## Figures and Tables

**Table 1:** Overview of the main implicit techniques used in human behaviour research.

Implicit Technique	Biometric Signal Measured	Sensor	Features	Psychological or Behavioural Construct Inferred
EDA (electro dermal activity)	Changes in skin conductance	Electrodes attached to fingers, palms or soles	Skin conductance response, tonic activity and phasic activity	Attention and arousal [80]
HRV (heart rate variability)	Variability in heart contraction intervals	Electrodes attached to chest or limbs or optical sensor attached to finger, toe or earlobe	Time domain, frequency domain, non-linear domain	Stress, anxiety, arousal and valence [81,82]
EEG (electroencephalogram)	Changes in electrical activity of the brain	Electrodes placed on scalp	Frequency band power, functional connectivity, event-related potentials	Attention, mental workload, drowsiness, fatigue, arousal and valence [83,84]
fMRI (functional magnetic resonance imaging)	Concentrations of oxygenated vs. deoxygenated haemoglobin in the blood vessels of the brain	Magnetic resonance signal	blood-oxygen-level dependent	Motor execution, attention, memory, pain, anxiety, hunger, fear, arousal and valence [85]
fNIRS (functional near-infrared spectroscopy)	Concentrations of oxygenated vs. deoxygenated haemoglobin in the blood	Near-infrared light placed on scalp	blood-oxygen-level dependent	Motor execution, cognitive task (mental arithmetic), decision-making and valence [86]
ET (eye-tracking)	Corneal reflection and pupil dilation	Infrared cameras point towards eyes	Eye movements (gaze, fixation, saccades), blinks, pupil dilation	Visual attention, engagement, drowsiness and fatigue [87]
FEA (facial expression analysis)	Activity of facial muscles	Camera points towards face	Position and orientation of head. Activation of action units	Basic emotions, engagement, arousal and valence [88]
SER (speech emotion recognition)	Voice	Microphone	Prosodic and spectral features	Stress, basic emotions, arousal and valence [89]

**Table 2:** Summary of previous research.

No	Author	Emotion	Signals	Features	Data Analysis	Subjects	HMD	VR Stimuli	Stimuli Comparison	Dataset Availability
1	Jang et al. (2002) [104]	Arousal	HRV, EDA	HR, HRV frequency domain, SCL, ST	*t*-test	11	VFX3D	3D flying and driving simulator	No	No
2	Meehan et al. (2005) [107]	Arousal	HRV, EDA	HR, SC, ST	*t*-test	67	Not reported	3D training room vs. pit room	No	No
3	Wilhelm et al. (2005) [108]	Anxiety	HRV, EDA	HR, SC	ANOVA, correlations	86	Not reported	3D height exposure	Partially (with a different real dataset)	No
4	Gorini et al. (2010) [109]	Anxiety	HRV, EDA	HR, SC	ANOVA	30 (20 with food disorders)	Not reported	3D photo and real food catering	VR vs. photo vs. real	No
5	Philipp et al. (2012) [110]	Valence	EMG	EMG	ANOVA	49	Virtual Research V8	3D room with IAPS pictures projected	No	No
6	Parsons et al. (2013) [111]	Arousal	HRV, EDA	HR, SC	ANOVA	50	eMagin Z800	3D high-mobility wheeled vehicle with Stroop task	No	No
7	Pallavicini et al. (2013) [112]	Stress	HRV, EMG, RSP	HR, SC, RR	ANOVA	39	Vuzix VR Bundle	3D classroom	No	No
8	Peperkorn et al. (2014) [43]	Fear	HRV, EDA	HR, SC	ANOVA	96 (48 spider-phobic)	eMagin Z800	3D virtual lab with time-varying threat (spiders and snakes)	No	No
9	Felnhofer et al. (2014) [113]	Anxiety	HRV	HR	ANOVA	75 (30 high anxiety)	eMagin Z800	3D lecture hall	No	No
10	Hartanto et al. (2014) [114]	Stress	HRV	HR	MANOVA	24 healthy subjects	eMagin Z800	3D stressful social environment	No	No
11	McCall et al. (2015) [115]	Arousal	HRV, EDA	HR, SC	Cross-correlations	306	NVIS nVisor SX60	3D room with time-varying threat (explosions, spiders, gunshots, etc.)	No	No
12	Felnhofer et al. (2015) [54]	Arousal	EDA	SCL	ANOVA	120	Sony HMZ-T1 3D	3D park with 5 variations (joy, sadness, boredom, anger and anxiety)	No	No
13	Notzon et al. (2015) [116]	Anxiety	HRV, EDA	HR, SC	ANOVA	83 (42 spider-phobic)	eMagin Z800	3D virtual lab with spiders	No	No
14	Hildebrandt et al. (2016) [117]	Arousal	HRV, EDA	RMSSD, SC	Regression	300	NVIS nVisor SX60	3D room with time-varying threats (explosions, spiders, gunshots, etc.)	No	No
15	Higuera-Trujillo et al. (2016) [118]	Stress	EDA	SCR	Kruskall–Wallis Test and correlations	12	Oculus Rift DK2	3D rooms (neutral, stress and calm)	No	No
16	Bian et al. (2016) [119]	Arousal	HRV, EMG, RSP	HR, LF, HF, LF/HF, RR, RS	Regression	36	Oculus Rift DK2	3D Flight simulator	No	No
17	Shiban et al. (2016) [120]	Stress	HRV, EDA	HR, SC	ANOVA	45	NVIS nVisor SX60	3D Trier Social Stress Test	No	No
18	Chirico et al. (2017) [121]	Awe	HRV, EDA, EMG	HF, VLF, SC	ANOVA	42	Samsung Gear VR	360° neutral and awe videos	Immersive vs. non-immersive	No
19	Zou et al. (2017) [122]	Arousal	HRV, EDA	HRV time domain (AVNN, SDNN…) and frequency domain (LF, HF…), SC, SCL, SCR	*t*-test	40	Oculus Rift DK2	3D fire evacuation	No	No
20	Breuninger et al. (2017) [123]	Arousal	HRV, EDA	HR, HF, SC	*t*-test	51 (23 agoraphobics)	TriVisio VR Vision	3D car accident	No	No
21	van’t Wout et al. (2017) [124]	Stress	EDA	SCR	MANOVA	44 veterans (19 with PTSD)	eMagin Z800	3D combat-related and classroom-related	No	No
22	Banaei et al. (2017) [125]	Arousal, Valence	EEG	PSD, ERSPs	MANOVA	17	Samsung Gear VR	3D rooms	No	No
23	Anderson et al. (2017) [126]	Stress	HRV, EDA	LF, HF, LF/HF, SC	MANOVA	18	Oculus Rift DK2	360° indoor vs. natural panoramas	No	No
24	Chittaro et al. (2017) [127]	Arousal	HRV	HR, LF, HF, LF/HF	ANOVA	108	Sony HMZ-T1 3D	3D cemetery and park	No	No
25	Higuera-Trujillo et al. (2017) [64]	Pleasantness	HRV, EDA	HF, SCR	Mann–Whitney U tests and correlations	100	Samsung Gear VR	3D, 360° and real retail store	real vs. 3D VR vs. 360° VR	No
26	Biedermann et al. (2017) [128]	Anxiety	HRV, EDA, RSP	HR, SC, RR	ANOVA	100	HTC Vive	Mixed reality (3D VR with real-world elements)	No	Yes
27	Tsai et al. (2018) [129]	Anxiety	HRV	HRV time domain (HR, RMSSD…) and frequency domain (HF, LF…)	ANOVA	30	eMagin Z800	3D VR claustrophobic environments	Augmented reality vs. VR	Upon request
28	Marín-Morales et al. (2018) [105]	Arousal, Valence	EEG, HRV	PSD and functional connectivity, HRV Time (HR, RMSSD…), frequency (HF, LF…) and non-linear (SD1, SD2, Entropy…) domain	SVM	60	Samsung Gear VR	360° virtual rooms	No	Upon request
29	Kisker et al. (2019) [130]	Arousal	HRV	HR	*t*-test, correlations and regressions	30	HTC Vive	3D exposure to a high height	No	No
30	Gromer et al. (2019) [131]	Fear	HRV, EDA	HR, SC	ANOVA	49 (height-fearful)	HTC Vive	3D forest	No	Yes
31	Zimmer et al. (2019) [132]	Stress	HRV, salivary	HR, salivary cortisol responses, salivary alpha amylase	ANOVA	50	Oculus Rift DK2	3D Trier Social Stress Test	Replication of a real study	No
32	Lin et al. (2019) [133]	Stress	EDA, Navigation	SC, travel distance, travel time	Mann–Whitney U	60	HTC Vive	3D, building on fire	No	No
33	Schweizer et al. (2019) [134]	Stress	HRV, EDA	HR, SC	*t*-test and correlations	80	TriVisio VR Vision	3D neutral and trauma-related scene	No	No
34	Kim et al. (2019) [135]	Calm, sadness and joy	Gait Patterns	Step count, gait speed, foot plantar pressure	ANOVA	12	HTC Vive	360° emotion-related videos	No	No
35	Uhm et at. (2019) [136]	Arousal	EEG	PSD	MANOVA	28	Samsung Gear VR	360° sport videos	No	No
36	Takac et al. (2019) [137]	Anxiety	HRV	HR	ANOVA	19	Oculus Rift	3D rooms with public audience	No	No
37	Marín-Morales et al. (2019) [65]	Arousal, Valence	HRV, EEG	PSD and functional connectivity, HRV Time (HR, RMSSD…), frequency (HF, LF…) and non-linear (SD1, SD2, Entropy…) domain	SVM	60	HTC Vive	3D art museum	Real museum vs. 3D museum	Upon request
38	Stolz et al. (2019) [138]	Fear	EEG	ERPs	ANOVA	29	Oculus Rift	3D room with angry avatars	No	No
39	Granato et al. (2020) [139]	Arousal, Valence	HRV, EDA, EMG, RSP	HR, SC, SCL, SCR, EMG, RR	SVM, RF, Gradient Boosting, Gaussian Process Regression	33	Oculus Rift DK2	3D video games	No	Yes
40	Bălan et al. (2020) [140]	Fear	HRV, EDA, EEG	HR, SC, PSD	kNN, SVM, RF, LDA, NN	8	HTC Vive	3D acrophobia game	No	No
41	Reichenberger et al. (2020) [141]	Fear	Eye-tracking	Fixation counts, TTFF	ANOVA, *t*-test	53 (26 socially anxious)	HTC Vive	3D room with angry avatars	No	Upon request
42	Huang et al. (2020) [142]	Stress	EDA	SCL	MANOVA	89	Oculus Rift DK2	360° built vs. natural environments	No	Yes

Signals: electroencephalograph (EEG), heart rate variability (HRV), electrodermalactivity (EDA), respiration (RSP) and electromyography (EMG). Features: heart rate (HR), high frequency (HF), low frequency (LF), LF/HF (low/high frequency ratio), very low frequency (VLF), total skin conductance (SC), skin conductance tonic level (SCL), fast varying phasic activity (SCR), skin temperature (ST), respiratory rate (RR), respiratory depth (RS), power spectral density (PSD), event-related spectral perturbations (ERSPs), event-related potencials (ERPs) and time to first fixation (TTFF). Data analysis: support vector machines (SVM), k-nearest neighbors algorithm (kNN), random forest (RF), linear discriminant analysis (LDA) and neural networks (NN).

**Table 3:** Previous research that included analyses of the validation of virtual reality (VR).

Type of Validation	% of Papers	Number of Papers
No validation	83.33%	35
Real	7.14%	3
Format	7.14%	3
Immersivity	2.38%	1
Previous datasets	2.38%	1
Replication	2.38%	1
